# Does the Type of Resin Luting Material Affect the Bonding of CAD/CAM Materials to Dentin?

**DOI:** 10.3390/dj13010041

**Published:** 2025-01-19

**Authors:** Burcu Dikici, Elif Türkeş Başaran, Esra Can

**Affiliations:** Department of Restorative Dentistry, Faculty of Dentistry, Yeditepe University, İstanbul 34728, Turkey; elif.turkes@yeditepe.edu.tr (E.T.B.); esra.cansay@yeditepe.edu.tr (E.C.)

**Keywords:** CAD/CAM, luting material, heavily filled flowable composite, bond strength, microhardness

## Abstract

**Background/Objectives:** This study aimed to investigate the microtensile bond strength (µTBS) of composite-based (Cerasmart), polymer-infiltrated (Vita Enamic), and feldspathic (Cerec) CAD/CAM blocks luted to dentin using a dual-cure resin cement (LinkForce), as well as micro-hybrid (G-aenial) and flowable composites (G-aenial Universal Flo), and evaluate the microhardness (HV) of luting materials through the CAD/CAM blocks. **Methods:** Cerasmart, Enamic, and Cerec were luted to dentin using three luting materials; LinkForce, G-aenial, and Universal Flo (n = 5). For HV, 117 disk-shaped specimens from LinkForce, G-aenial, and Universal Flo (n = 13) were polymerized through 3 mm thick CAD/CAM. Thirty-nine light-cured specimens without CAD/CAM were used as control. Following 24 h storage, the µTBS and HV were evaluated. Data were analyzed using the two-way ANOVA and post hoc Tukey tests (*p* < 0.05). **Results:** The µTBS to dentin and HV were significantly influenced by the type of luting material and CAD/CAM material. With all the CAD/CAM materials, LinkForce and Universal Flo exhibited a significantly similar µTBS to that of dentin (*p* > 0.05). Compared with the control group, all the HV values of the luting materials decreased significantly (*p* < 0.05). **Conclusions:** Heavily filled flowable composites exhibit a bonding effectiveness similar to that of dual-cure resin cements. All the luting materials showed similar HV when polymerized through the polymer-infiltrated CAD/CAM material.

## 1. Introduction

Failures in adhesively bonded indirect restorations can occur due to biological factors such as secondary caries, pulp pathology, and chronic and acute apical periodontitis, as well as due to mechanical factors such as tooth and root fractures, the chipping of ceramic and resin composite materials, or the loss of retention and adhesion [1]. Mechanical problems related to the restorative materials depend on the material’s properties, while retention loss and adhesion problems are associated with bonding between the resin luting–tooth structure and resin-luting-restorative material interfaces [2]. In this bonded assembly, each parameter, such as the adhesive approach, resin-luting material, and the restorative material’s properties, plays a crucial role in restoration durability. With regard to the adhesives and the adhesive approach, a recent meta-analysis and systematic review revealed that self-etch and etch-and-rinse adhesives did not significantly differ in survival rates, whereas self-adhesive luting resins showed a significantly lower survival rate than both selective enamel-etch and self-etch adhesive approaches in adhesively bonded indirect restorations [3]. With respect to indirect restorative materials, since their introduction in 1985, CAD/CAM technology has facilitated the development of various materials, such as lithium disilicate glass–ceramics, hybrid ceramics, feldspathic ceramics, and polymer-infiltrated ceramics, which can be adhesively bonded to tooth structures. Glass–ceramic CAD/CAM materials are prominent owing to their advantages such as high strength, color stability, adequate esthetics, low surface roughness, biocompatibility, and minimal wear against chewing forces [4], whereas composites, hybrid ceramics, and polymer-infiltrated ceramics have similar elastic moduli to dentin, easy machinability, easy finishing and repair procedures, and less wear against opposing teeth [4,5,6]. The translucency of the CAD/CAM materials, which varies depending on its microstructure, composition, shade, size, and restoration thickness [7,8] also affects the clinical performance of indirect restorations, as it has a major impact on the resin luting material’s mechanical properties and bonding performance to dentin [7].

Regarding the luting materials, dual-cure resin cements are the preferred materials in adhesively bonded indirect restorations, combining chemical and light polymerizing properties and achieving continuous polymerization in the absence of light activation or under thick restorations [9]. Although the shade, monomer composition and filler properties of the resin luting materials are crucial factors in the polymerization process [10], insufficient marginal integrity, water degradation over time, increased wear, and high polymerization shrinkage are the drawbacks of dual-cure resin cements. Several in vivo [11,12] and in vitro studies [13,14,15] suggested using preheated or non-heated light-cure resin composites as an alternative for bonding indirect adhesive restorations to dental hard tissues instead of light-cure and dual-cure luting cements, owing to their excellent mechanical properties, handling characteristics, and wear resistance [16]. In fact, a recent systematic review suggested using light-cure resin composites for luting translucent and thin CAD/CAM restorations [17]. However, the degree of conversion and depth of cure of light-cure composites under thick indirect restorations remain a concern with respect to the durability and biocompatibility of the restorations [18,19]. 

Thus, this in vitro study aimed to investigate the microtensile bond strengths (µTBS) of composite-based, polymer-infiltrated, and feldspathic ceramic CAD/CAM blocks bonded to dentin using dual-cure resin cement, a light-cure paste-type composite, and heavily filled flowable composite, and to assess the microhardness (HV) of these resin luting materials polymerized through CAD/CAM blocks. The null hypotheses tested were:

1. CAD/CAM blocks do not affect the adhesion of different resin luting materials to dentin.
2. CAD/CAM blocks do not affect the HV of different resin luting materials.
3. The µTBS of the light-cure paste-type composite and heavily filled flowable composite to dentin is similar to the dual-cure resin cement.
4. The HV of the light-cure paste-type composite and heavily filled flowable composite is similar to the dual-cure resin cement.

## 2. Materials and Methods

This study was approved by the Research Ethics Committee of Yeditepe University (No. 1418). The compositions and manufacturers of the materials are shown in Table 1.

### 2.1. Selection and Preparation of the Teeth for µTBS

Forty-five non-carious human third molars that were extracted within 3 months were selected for the study. The remaining hard and soft tissues were cleaned with periodontal curettes and kept in distilled water with 0.1% thymol solution. The sample size was determined using power analysis (G*Power 3.1 Software; Germany) and was determined as 5 teeth for µTBS testing and 10 blocks for HV testing to reach a medium effect size with 80% power and a significance level of 0.05. 

After the removal of the enamel with a diamond saw (IsoMet, Buehler Ltd., Lake Bluff, IL, USA), dentin surfaces of the teeth were prepared using 600 grit silicon carbide paper (SiC) with a polishing machine (Buehler Ltd., IL, USA) under water cooling. Standardize the smear layer were obtained with 2–3 mm remaining dentin thickness. The roots of the teeth were also removed 2 mm below the cemento-enamel junction with a diamond saw. Based on the CAD/CAM material used, the teeth were allocated into the following three groups (n = 15 each): Cerasmart (GC, Tokyo Japan), Vita Enamic (Vita Zahnfabric, Bad Säckingen, Germany), and Cerec (Dentsply Sirona, Bensheim, Germany). For adhesive cementation, each CAD/CAM block was luted using three different luting materials (n = 5 specimens of each CAD/CAM per luting material): LinkForce (GC, Tokyo, Japan), G-aenial composite (GC, Tokyo, Japan), and G-aenial Universal Flo (GC, Tokyo, Japan).

The 3 mm thick slabs were obtained from the tested rectangular-shaped blocks with a diamond saw (IsoMet, Buehler Ltd., Lake Bluff, IL, USA) and polished at 400 Rpm (Buehler Ltd., IL, USA). The thickness of all slabs was checked with a digital caliper. The intaglio surfaces of Cerasmart slabs were sandblasted (Basic Classic, Hilzingen, Germany). Sandblasting was conducted with 50 µm aluminum oxide particles (Al_2_O_3_) at 0.2 MPa pressure and 10 mm distance for 10 s. Following the application of 5% hydrofluoric acid (Ultradent Porcelain Etch; Ultradent Product Inc., South Jordan, UT, USA) to Enamic and Cerec surfaces for 60 s, they were rinsed for 60 s with a water spray. Subsequently, all the slabs underwent 5 min of ultrasonic cleaning. After applying G-multi-Primer (GC, Tokyo, Japan) to the intaglio surfaces of all the tested blocks in accordance with the manufacturer’s recommendations, G-Premio Bond (GPB, GC, Tokyo, Japan) was applied and the blocks were polymerized for 10 s (Demi Ultra, 1100 mW/cm^2^; Kerr Dental, CA, USA). Throughout the luting process, a radiometer was used to verify the output of the light intensity.

To ensure that the resin luting material’s thickness was uniform and standard, 100 µm thick adhesive tapes were applied to the dentin surfaces. The universal adhesive (GPB) was applied to the dentin surfaces in self-etch mode for 10 s, air-thinned for 5 s, and polymerized for 10 s (Demi Ultra). Subsequently, the Cerasmart, Enamic, and Cerec specimens were luted to the dentin surfaces using LinkForce, G-aenial composite, and G-aenial Universal Flo. Excessive resin luting material was removed from around the CAD/CAM block and dentin surface. A light-curing unit was positioned 1 mm away from the surface and all the luted specimens were instantly polymerized on all proximal sides and then, finally, light-cured from the top surfaces for 20 s each at a seating force of 1 kg [21], which was applied continuously for five minutes to ensure the complete polymerization of the resin luting material. 

All specimens were kept in distilled water at 37 °C for 24 h. The CAD/CAM–resin–dentin blocks were then sectioned into 1 mm × 1 mm sticks with a diamond saw (IsoMet, Buehler Ltd., Lake Bluff, IL, USA). The dimensions of each beam were checked using a digital caliper with ±0.1 mm accuracy. Sticks from each tooth’s center area were used, while the sticks from the periphery were not utilized for the study. A digital caliper was used for the measurement of the remaining dentin thickness. Specimens with 2–3 mm dentin thickness were used. Each stick was fixed to a customized metallic attachment using cyanoacrylate adhesive (Pattex, Henkel AG &Co, Düsseldorf, Germany). The sticks from each group (n = 25) were subjected to µTBS testing using a universal testing machine (Instron, Norwood, MA, USA) at a crosshead speed of 0.5 mm/s [22].

The failure types of the fractured specimens were assessed with a stereomicroscope (Leica MZ16 FA, Gantenbein, Switzerland) and were categorized as adhesive, mixed, and cohesive. Adhesive failures occurred between dentin and the resin luting material, mixed failures between the resin luting material and the dentin, accompanied by part of the luting material remaining on the CAD/CAM, and cohesive failures within the CAD/CAM, dentin, or resin luting material [23]. Selected specimens with the most observed failures and close to the mean µTBS value of the respective groups were analyzed using a scanning electron microscope (SEM) (6335-F, JEOL Ltd., Tokyo, Japan) following a standard procedure, including fixation, dehydration, chemical drying, and gold sputter-coating [24]. 

### 2.2. Specimen Preparation and Evaluation of the HV 

To assess the HV of the resin luting materials (control groups), 39 disk-shaped specimens (2 mm thickness, 5 mm diameter) were prepared from LinkForce, G-aenial, and Universal Flo. The materials were placed inside a circular metallic mold, covered with a transparent Mylar strip, compressed with microscopic glass slides (1 mm thickness) from both sides and light-cured for 40 s. Then, the specimens were polished using 800-1200-4000 grit SiC paper under water cooling prior to the HV measurement. 

With respect to the HV evaluation of the resin luting materials polymerized through CAD/CAM blocks, Cerasmart, Enamic, and Cerec blocks were sectioned in 3 mm thick slabs (N = 117, n = 39 from each CAD/CAM block) and the intaglio surfaces of the slabs were pretreated as described in the luting procedure to dentin. A transparent Mylar strip was placed over the resin luting materials, which were applied in the metallic molds as in the control groups, and the respective 3 mm thick CAD/CAM slabs were positioned in contact with them. Then, the light-curing unit was placed on the CAD/CAM slabs and polymerized for 40 s (n = 13 per group). All procedures were performed in a dark chamber to prevent additional polymerization; then, the specimens were stored in the dark chamber at 37 °C for 24 h [25].

HV was then evaluated using a Vickers microhardness indenter (Buehler Ltd., IL, USA) with a 50 g load for 15 s. Three indentations were performed on the surface of each specimen, which were evenly distributed around in a circle, no closer than 0.5 mm to the neighboring indentations, and a mean value was calculated from these three evaluations. Thirteen mean HV values for each group were then used for the statistical analysis.

### 2.3. Statistical Analysis

In order to assess the data, the Shapiro–Wilk normality test was used to check the variables’ distribution in addition to descriptive statistical techniques like mean and standard deviation. The µTBS and HV data were analyzed using the two-way analysis of variance and post hoc Tukey test, with the type of CAD/CAM blocks and luting material serving as independent variables (NCSS 2007 Statistical Software, NCSS, USA). Statistical significance was set at *p*-value < 0.05 for all tests.

## 3. Results

The mean and standard deviations of the µTBS values (MPa) of different resin luting materials to dentin are presented in Table 2. µTBS to dentin was significantly affected by the type of luting material (*p* < 0.0001) and CAD/CAM material (*p* < 0.0001). No significant interaction was observed between the type of CAD/CAM and luting material (*p* > 0.05).

Regarding Enamic, the µTBS to dentin was significantly higher with LinkForce (31.56 ± 2.74 MPa) and UniversalFlo (33.73 ± 4.16 MPa) than with G-aenial (27.09 ± 2.89 MPa) (*p* < 0.005), and the difference between LinkForce and Universal Flo was not significant (*p* > 0.05). With respect to Cerasmart, a significant difference in the luting material’s µTBS to dentin was observed between Universal Flo (30.78 ± 3.08 MPa) and G-aenial (26.53 ± 4.16 MPa) (*p* < 0.05), whereas no statistically significant differences were observed between LinkForce and Universal Flo and between LinkForce and G-aenial (*p* > 0.05). As for Cerec, no significant differences were observed in the µTBS of all the luting materials to dentin. The µTBS with Universal Flo (28.23 ± 3.61 MPa) was similar to that with G-aenial (24.90 ± 3.09 MPa) and LinkForce (27.07 ± 3.92 MPa) (*p* > 0.05). 

When the µTBS values with the same luting material for different CAD/CAM blocks were compared, Universal Flo showed a statistically similar µTBS to dentin in the Cerasmart, Enamic, and Cerec groups (*p* > 0.05). Similarly, no statistically significant differences were observed with G-aenial among the Cerasmart, Enamic, and Cerec groups (*p* > 0.05). On the other hand, LinkForce resulted in significantly higher µTBS to dentin with Enamic than with Cerec (*p* = 0.017); however, the differences between Cerasmart and Enamic and between Cerasmart and Cerec were not statistically significant (*p* > 0.05) (Table 2). 

The means and standard deviations of the HV values of the resin luting materials are listed in Table 3. HV was significantly affected by the type of luting material (*p* < 0.0001) and CAD/CAM blocks (*p* < 0.0001). All luting materials’ HV values decreased significantly when polymerized under the CAD/CAM blocks (*p* < 0.05); thus, the HV of control groups for all tested CAD/CAM materials showed significantly higher values when compared to CAD/CAM groups (*p* < 0.05). The HV was significantly higher for Universal Flo (57.97 ± 3.05) and G-aenial (56.93 ± 3.84) than for LinkForce (47.04 ± 2.08) in the control group.

When polymerized through Cerasmart and Cerec, no significant differences were found among the HV values of LinkForce, G-aenial, and Universal Flo (*p* > 0.05). On the other hand, when polymerized through Enamic, the HV was significantly lower for LinkForce (36.96 ± 2.88) than for Universal Flo (40.17 ± 2.47) and G-aenial (41.90 ± 2.62 HV), which were not significantly different (*p* > 0.05). For each resin luting material polymerized through Cerasmart, Enamic, and Cerec, no significant differences were evaluated in HV values (*p* > 0.05) (Table 3).

**Figure 1:**
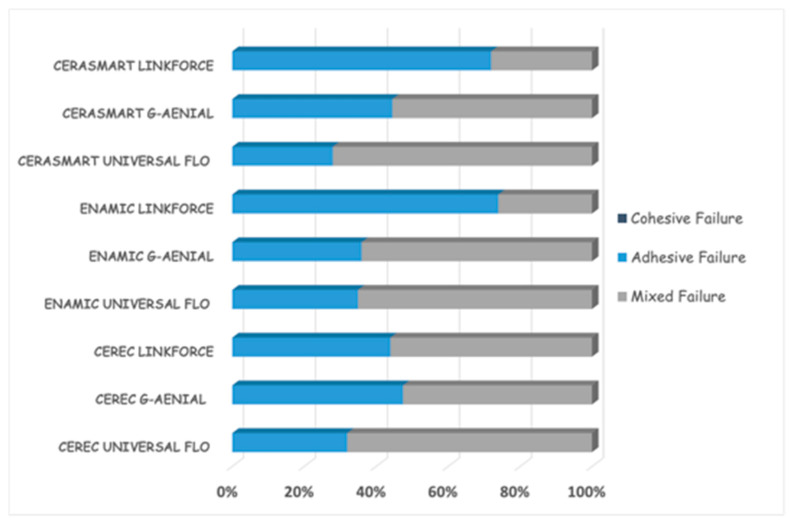
Failure mode analysis of fractured specimens.

**Figure 2:**
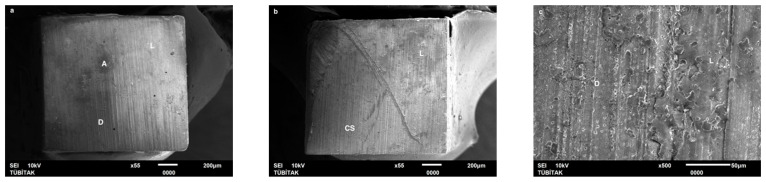
(**a**–**c**): Cerasmart/Linkforce: Scanning electron micrographs of Cerasmart luted with LinkForce. Adhesive failure occurred between the dentin and resin cement. CS: Cerasmart, A: adhesive, D: dentin, L: LinkForce.

**Figure 3:**
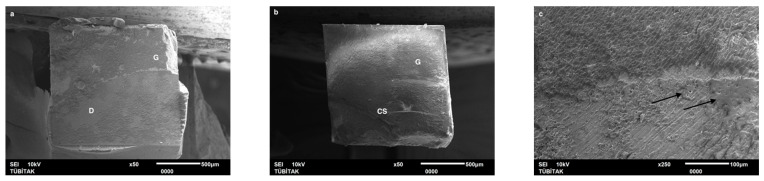
(**a**–**c**): Cerasmart/G-aenial: Scanning electron micrographs of Cerasmart luted with G-aenial. Mixed failure occurred between the dentin and composite with numerous voids. CS: Cerasmart, A: adhesive, D: dentin, G: G-aenial. The arrows indicate voids.

**Figure 4:**
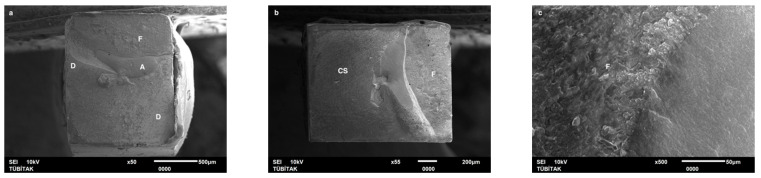
(**a**–**c**): Cerasmart/Universal Flo: Scanning electron micrographs of Cerasmart luted with Universal Flo, showing mixed failure between the CAD/CAM material, heavily filled flowable composite, and dentin. CS: Cerasmart, A: adhesive, D: dentin, F: Universal Flo.

**Figure 5:**
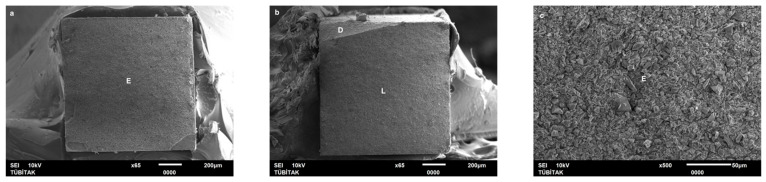
(**a**–**c**): Enamic/LinkForce: Scanning electron micrographs of Enamic luted with LinkForce, revealing adhesive failure between the CAD/CAM material and resin cement. E: Enamic, A: adhesive, D: dentin, L: LinkForce.

**Figure 6:**
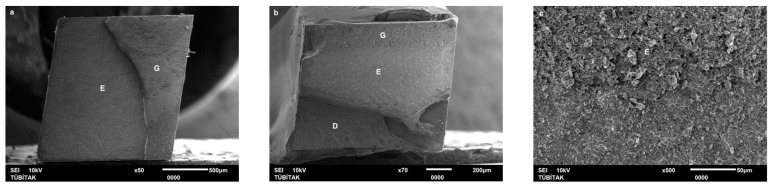
(**a**–**c**): Enamic/G-aenial: Scanning electron micrographs of Enamic luted with G-aenial. Mixed failure occurred between the CAD/CAM material, composite, and dentin. E: Enamic, A: adhesive, D: dentin, G: G-aenial.

**Figure 7:**
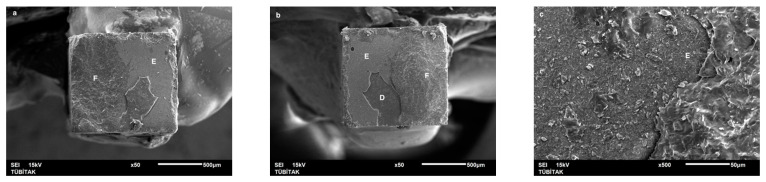
(**a**–**c**): Enamic/Universal Flo: Scanning electron micrographs of Enamic luted with Universal Flo, showing mixed failure between the CAD/CAM material, heavily filled flowable composite, and dentin. E: Enamic, A: adhesive, D: dentin, F: Universal Flo.

**Figure 8:**
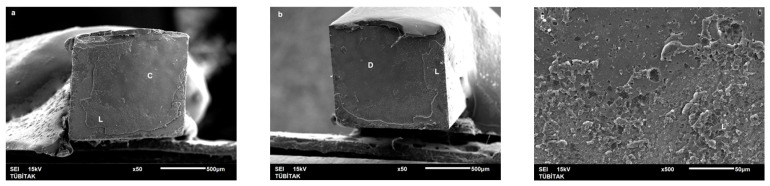
(**a**–**c**): Cerec/LinkForce: Scanning electron micrographs of Cerec luted with LinkForce, revealing mixed failure between the CAD/CAM material, dentin, and resin cement. C: Cerec, A: adhesive, D: dentin, L: LinkForce.

**Figure 9:**
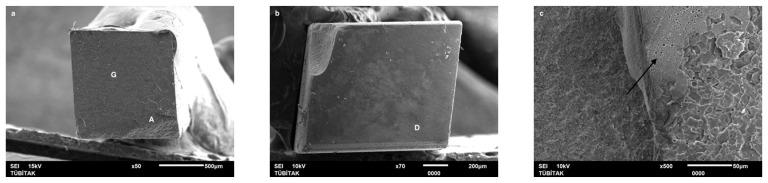
(**a**–**c**): Cerec/G-aenial: Scanning electron micrographs of Cerec luted with G-aenial. Mixed failure occurred between the CAD/CAM material, composite, and dentin, showing numerous voids. C: Cerec, A: adhesive, D: dentin, G: G-aenial. The arrow indicates a void.

**Figure 10:**
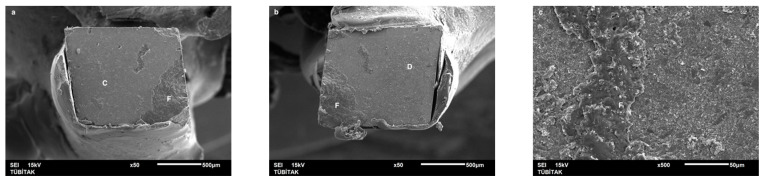
(**a**–**c**): Cerec/Universal Flo: Scanning electron micrographs of Cerec luted with Universal Flo, revealing mixed failure between the CAD/CAM material, heavily filled flowable composite, and dentin. C: Cerec, A: adhesive, D: dentin, F: Universal Flo.

Failure mode distribution following µTBS testing is shown in Figure 1. According to the failure analysis, all the specimens exhibited adhesive and mixed fractures. Cohesive fractures in the dentin, CAD/CAM blocks, or resin luting material were not evident. SEM images of the Cerasmart, Enamic, and Cerec slabs with all the resin luting materials are shown in Figure 2, Figure 3, Figure 4, Figure 5, Figure 6, Figure 7, Figure 8, Figure 9 and Figure 10, respectively. The failure modes of Cerasmart luted with LinkForce mostly showed adhesive failure between the dentin and the luting material (Figure 2a–c), whereas G-aenial and Universal Flo showed mixed failures between the luting material, dentin, and CAD/CAM (Figure 3a–c and Figure 4a–c, respectively). Enamic luted with G-aenial and Universal Flo showed a mixed failure pattern between the CAD/CAM block, dentin, and luting material (Figure 6a–c and Figure 7a–c); however, LinkForce resulted mostly in adhesive failure between the luting material and CAD/CAM (Figure 5a–c). A failure mode analysis of Cerec revealed mixed failure patterns for all the resin luting materials (Figure 8a–c, Figure 9a–c and Figure 10a–c). 

## 4. Discussion

This in vitro study aimed to investigate the µTBS of Cerasmart, Enamic, and Cerec CAD/CAM blocks luted to dentin using LinkForce, G-aenial, and Universal Flo, and to evaluate the microhardness (HV) of these resin luting materials polymerized through the CAD/CAM blocks.

The μTBS test is a reliable method testing the bonding effectiveness of different restorative materials to dentin [26]. Along with structural differences in dentin, the bonding performance of indirect restorative materials to dentin is affected by the luting materials’ mechanical properties and the HV test provides insight into the degree of polymerization, which is directly related to the mechanical properties of the luting materials under different restorative materials [27,28,29]. Therefore, in this study, the bonding between the CAD/CAM blocks and dentin with different resin luting materials was investigated using the μTBS test and the depth of cure of these luting materials through the CAD/CAM blocks was evaluated using the HV test.

The translucency, thickness, and composition of restorative materials have a significant impact on the light transmittance through indirect restorations, resulting in low-light irradiance towards the luting material [30,31]. Because the light attenuation overlying a ceramic thickness above 3 mm adversely affected the polymerization of luting materials, a ceramic thickness of 3 mm was considered the critical threshold [8,32]. Therefore, in this study, highly translucent CAD/CAM materials with a thickness of 3 mm were used to promote increased light transmission and polymerization of the resin luting materials. Furthermore, all polymerization procedures were performed using a high-intensity light curing unit (1100 mW/cm^2^) with an extended irradiation time, as recommended in the literature, to ensure effective curing of the luting materials [33].

According to the results of the µTBS test, the adhesion of different resin luting materials to dentin was significantly influenced by the type of CAD/CAM block (*p <* 0.001), thus rejecting the first null hypothesis that CAD/CAM blocks do not affect the adhesion of the luting materials to dentin. Enamic exhibited a significantly higher µTBS than Cerec with LinkForce and Universal Flo, whereas the differences between Cerasmart and Enamic and between Cerasmart and Cerec were not significant (*p* > 0.05). The adhesion performance and translucency of CAD/CAM materials are significantly affected by their intaglio surface pretreatment techniques [34]. While HF is frequently recommended for achieving adhesion to glass–ceramic materials, 50 μm Al_2_O_3_ sandblasting is typically advised for composite CAD/CAM blocks to achieve micromechanical retention [20]. However, for polymer-infiltrated ceramic, both HF and sandblasting are indicated [35]. In this study, HF was used for Enamic and Cerec, which preferentially dissolves the glassy matrix; thus, the higher µTBS of Enamic than that of Cerec with LinkForce and Universal Flo could be attributed to the micromechanical retention created by HF etching and the removal of the organic matrix of Enamic that created porosities up to 10 μm deep. This microstructure may have favored the adhesion of the bonding agents/luting materials to the surface. Conversely, Enamic shows less translucency because of its high alumina concentration (8.31 wt%) that causes lower translucency compared to Cerasmart, which has a lower percentage of alumina incorporated into a firmly cross-linked resin matrix [36]. The inorganic matrix of Cerasmart is composed of nanoceramics reinforced by zirconia; for surface pretreatment, it was sandblasted with Al_2_O_3_, which may have decreased the translucency of the materials [37] due to some fillers functioning as radiopacifiers, and their excessive addition at higher concentrations alters the material’s translucency [37]. This may explain the statistically similar µTBS of Enamic and Cerasmart with all the tested resin luting materials. Consistent with our results, Lise et al. [13] also stated that the μTBS of a self-adhesive resin cement and Universal Flo to Enamic was statistically similar to that of Cerasmart before and after aging. 

Similarly to the µTBS, different types of CAD/CAM materials also had a significant effect on the HV of the resin luting materials (*p* > 0.05), rejecting the second null hypothesis that the CAD/CAM blocks do not affect the HV of the resin luting materials. Ceramics with a high crystalline content tend to be less translucent [38]. Feldspathic ceramics provide improved light transmission owing to their high vitreous phase content and low crystalline phase concentration [36]. The higher translucency of the feldspathic ceramic Cerec compared to the polymer-infiltrated Enamic could have a positive effect on the HV of the luting materials; however, the results of this study showed no significant differences between the HV of the resin luting materials polymerized through Cerec and Enamic. As mentioned previously, Enamic showed less translucency owing to its high alumina content compared to Cerasmart [35]. However, no significant differences were observed between Cerasmart and Enamic for all the tested luting materials, indicating that despite the differences in microstructures, equal polymerization efficiency is demonstrated by the polymerization of various resin luting materials through the 3 mm thick CAD/CAM blocks of composite-based, polymer-infiltrated ceramic and feldspathic ceramic. 

Despite the widespread use of dual-cure resin cements for cementing indirect bonded restorations, concerns regarding their clinical performance persist, such as marginal discoloration, fractures and secondary caries owing to their low filler content, polymerization problems, and poor mechanical properties [30]. Therefore, the use of heated and unheated light-cured composites is recommended to improve the success performance of bonded indirect restorations, advised to be investigated in in vitro and in vivo studies [39,40]. Recently, heavily filled flowable composites with improved mechanical properties and enhanced clinical indications have been developed [39,41]. The benefit of flowable composites over resin cements is that they combine a higher filler content with low viscosity, which enhances the mechanical qualities [42] and contributes to the longevity of the restoration. Compared to paste-type composites, these materials are less viscous, which improves their adaptability to the cavity walls and margins and creates fewer voids after luting with high monomer conversion [43]. Additionally, paste-type composites may cause improper seating of the indirect restorations owing to their high viscosity, exacerbating the consequences of thermal expansion and shrinkage during polymerization [44]. In this study, LinkForce and Universal Flo showed statistically similar µTBS with all the tested CAD/CAM materials to dentin; however, G-aenial resulted in significantly lower µTBS than LinkForce and Universal Flo for Enamic and Cerasmart and lowest for Cerec. Therefore, the third hypothesis that µTBS of light-cure paste-type composite and heavily filled flowable composite to dentin is similar to the dual-cure resin cement was rejected. Consistent with our results, Hassanien and Tolba [39] showed that using a flowable composite resulted in statistically similar bond strengths to those achieved between a resin cement and Enamic and a composite CAD/CAM block. Similarly, El-Askary et al. [45] reported that the µTBS of a composite CAD/CAM block to dentin was significantly higher when luted with a flowable composite than with a paste-type composite. In contrast to our findings, Grangeiro et al. [46] showed that a resin cement has a higher micro-shear bond strength than flowable composite to Enamic, with and without aging, while a preheated composite resulted in the highest bond strength. The difference in their results could be due to the differences between the µTBS and micro-shear bond strength testing protocols. Additionally, in this study, to mimic the clinical situation, CAD/CAM blocks were luted to dentin with different resin luting materials, whereas Grangeiro et al. [46] directly luted the resin cement, flowable composite, and preheated composite to the CAD/CAM blocks of 2 mm thickness, which resulted mostly in cohesive failures in the luting materials and ceramic CAD/CAM blocks. Moreover, this study did not evaluate preheating and G-aenial composite resulted in the lowest µTBS in all the CAD/CAM blocks, most probably due to the insufficient light penetration through the composite, which did not contribute to the additional polymerization of the thin universal adhesive on the dentin side that resulted in several voids caused by water inflow during storage that was shown in SEM images (Figure 3b and Figure 9b, respectively). As advised in the literature, preheating may decrease the viscosity of the composite, which facilitates the seating of the restoration; however, Liberato et al. [47] revealed that this procedure might also increase the tendency of shrinkage stress, which could result in the decreased bond strength of the resin luting material. With respect to the failure modes, G-aenial (Figure 3, Figure 6 and Figure 9) and Universal Flo (Figure 4, Figure 7 and Figure 10) resulted in mixed failures with all the CAD/CAM materials, while LinkForce showed predominantly adhesive failures between the resin cement and CAD/CAM–dentin interface in Cerasmart and Enamic (Figure 2 and Figure 5), even though Universal Flo and LinkForce resulted in similar µTBS to dentin in Cerasmart and Enamic. The bond strength of resin luting materials depends on several factors, including the mechanical properties of the material, the presence or absence of voids, or microstructural defects at the interface and its flowability [45]. 

With respect to HV, Universal Flo (57.97 ± 3.05) and G-aenial (56.93 ± 3.84) showed significantly higher HV values than LinkForce (47.04 ± 2.08), which was attributed to the higher filler ratio of G-aenial (77 wt%) and Universal Flo (69 wt%) compared to LinkForce (63 wt%) [20]. The cross-linkage type and filler content in the polymerized resin determine the mechanical characteristics and hardness of any resinous material. The strength of the material increases with increasing filler loading [48,49]; hence, both Universal Flo and G-aenial showed higher HV values than LinkForce. However, polymerization through the CAD/CAM materials resulted in a significant drop in the HV values for all evaluated resin luting materials (*p* < 0.05). Runnacles et al. [50] examined the conversion degree of light-cure resin cements beneath ceramics of varying thicknesses and found that the conversion degree decreased significantly at thicknesses measuring ≥1.5 mm. The degree of polymerization of resin materials has been evaluated using the HV as a criterion, due to the favorable correlation between the HV of a material and the degree of conversion of resin cement, as demonstrated by numerous studies [51,52,53]. Ozturk et al. [53] conducted an investigation into the polymerization rate and HV of resin luting cements utilized in ceramic restorations and discovered a positive correlation between the two variables. In this study, all the resin luting materials showed a significant decrease in the HV, which was attributed to the fact that the quantity of light that reaches the resin luting materials is influenced by the thickness of the CAD/CAM materials. LinkForce, G-aenial, and Universal Flo showed similar HV values through Cerasmart and Cerec; however, through Enamic, Universal Flo and G-aenial showed significantly higher HV values than LinkForce. Thus, the fourth hypothesis that the HV of light-cure paste-type and flowable composites through CAD/CAM blocks is similar to the dual-cure resin cement was rejected. Similarly to our study, Go et al. [54] reported that resin materials under 2 mm and 4 mm blocks demonstrated reduced HV because of the nonhomogeneous light energy that flowed through the CAD/CAM materials. This finding corroborates the findings of numerous previous studies, showing that the HV values of resin materials were lower through thick ceramic blocks than thinner ones, probably because lower energy levels reached the deeper layers of the resin materials [55,56]. In contrast, Kilinc et al. [57] assessed the dual-cure resin cements’ degrees of conversion using different thicknesses of ceramics (1–4 mm) and showed a similar degree of conversion up to 3.0 mm thickness. 

During the adhesive luting of indirect restorations to stabilize the hybrid layer and the adhesive interface, independent light curing of the adhesive on the dentin surface is advised [23,58]. According to Faria-E-Silva and Pfeifer [56], many universal adhesives actually form a thin layer, with an average thickness of 10 µm; theoretically, the restoration is luted to fit perfectly and should not be affected by the separate light curing of the adhesive. In the present study, the universal adhesive was light-cured on the dentin side and, according to the failure mode analysis, none of the specimens showed adhesive failure between dentin and resin luting materials, indicating that the weakest link in the bonding assembly was not at this interface. However, as discussed earlier, luting CAD/CAM blocks with the light-cure paste-type composite to dentin resulted in the lowest µTBS with all the tested materials, and numerous voids were evident in the adhesive layer, which may further negatively affect the adhesion to dentin when an aging protocol is applied.

The clinical relevance of this study is that the light-cure heavily filled flowable composite could be an alternative to the dual-cure resin cement as the resin luting material in 3 mm thickness CAD/CAM restorations with a controlled and prolonged working time, particularly in terms of controlled and extended working times to adjust the restoration during placement, ensuring more precise mechanical properties and minimizing the risk of premature curing. Given the increasing use of CAD/CAM technology in restorative dentistry, this study explores a promising approach to enhance the efficiency and predictability of luting procedures for ultimately better clinical outcomes. However, the limitations of this study include the use of only one type of restoration thickness and evaluating the bonding performance after 24 h without an aging protocol. Therefore, further in vitro research concentrating on the degree of conversion with different restoration thicknesses and long-term in vitro and in vivo adhesion studies are necessary to validate the results of this study.

## 5. Conclusions

Within the limitations of this study, the following conclusions can be made:

(1) The light-cure heavily filled flowable composite showed similar bonding performance to the dual-cure resin cement when bonding to dentin, for all the tested CAD/CAM materials.
(2) The light-cure paste-type composite showed the lowest bonding performance to dentin for all the tested CAD/CAM materials.
(3) When polymerized through different types of CAD/CAM materials, dual-cure resin cement, light-cure paste-type composite, and heavily filled flowable composite exhibited similar microhardness.

## Figures and Tables

**Table 1:** Materials used in this study [20].

Materials	Manufacturer	Type	Composition	Filler Ratio % by wt
**Cerasmart**	GC Corporation, Tokyo, Japan	Composite CAD/CAM block	Bis-MEPP, UDMA, DMA silicon dioxide (20 nm), barium glass (300 nm), nanoparticle-filled resin containing silica monomers	71% wt
**Vita Enamic**	Vita Zahnfabrik, Bad Sackingen, Germany	Polymer-infiltrated CAD/CAM block	Ceramic: silicon dioxide 58–63%, aluminum oxide 20–23%, sodium oxide 9–11%, potassium oxide 4–6%, boron trioxide 0.5–2%, zirconia and calcium oxide. Polymer part (25%): UDMA and TEGDMA	86% wt
**Cerec**	Dentsply Sirona	Feldspathic CAD/CAM block	Feldspathic crystalline particles in glassy matrix (SiO_2_ Al_2_O_3_ Na_2_O K_2_O CaO TiO_2_	
**Linkforce**	GC Corporation, Tokyo, Japan	Dual-cure resin cement	Paste A: bis-GMA UDMA, DMA, initiator, pigments; Paste B: bis-MEPP, UDMA, DMA, initiator, Bis-EMA, dibenzoyl peroxide, BHT barium borate glass	63% wt/38% vol
**G-aenial Posterior**	GC Corporation, Tokyo, Japan	Micro-hybrid composite	UDMA and dimethacrylate, bis-GMA free, pre-polymerized fillers (16–17 μ). Strontium and lanthanide fluoride. Silica and fluoroaluminosilicate > 100 nm, fumed silica < 100 nm.	77% wt/65% vol
**G-aenial Universal Flo**	GC Corporation, Tokyo, Japan	Heavily filled flowable composite	UDMA, Bis-MEPP, TEGDMA, silicon dioxide (16 nm) and strontium glass (200 nm) pigments, photoinitiator	69% wt/60% vol
**G-multi-** **primer**	GC Corporation, Tokyo, Japan	Ceramic primer	Ethyl alcohol, phosphoric acid ester, silane, MDP, MDTP, DMA	
**G-Premio Bond**	GC Corporation, Tokyo, Japan	GC Corp, Tokyo, Japan	10-MDP, 4-META, MDTP, methacrylate acid ester, distilled water, acetone, photo initiators, fine powdered silica	

Bis-MEPP: 2,2-Bis (4-methyacryloxypoly-ethoxyphenyl) propane, UDMA: urethane dimethacrylate, DMA: dodecyl dimethacrylate, TEGDMA: triethylene glycol dimethacrylate; Bis-GMA: bisphenol A glycidyl methacrylate; MDP:10-methacryloyloxydecyl dihydrogen phosphate; MDTP:10-methacryloyloxydecyl dihydrogen thiophosphate; 4-META: 4 methacryloxyethyl trimellitic anhydride. wt: weight; vol: volume.

**Table 2:** The mean and standard deviations of the µTBS values (MPa) of the resin luting materials to dentin.

	Linkforce	G-Aenial	Universal Flo
**CERASMART**	29.5 ± 4.45 ^a,c,A,C^	26.53 ± 4.16 ^b,c,A^	30.78 ± 3.08 ^a,A^
**VITA ENAMIC**	31.56 ± 2.74 ^a,A^	27.09 ± 2.89 ^b,A^	33.73 ± 4.16 ^a,A^
**CEREC**	27.07 ± 3.92 ^a,B,C^	24.90 ± 3.09 ^a,A^	28.23 ± 3.61 ^a,A^

Different uppercase letters in the same column indicate significant differences between different CAD/CAM materials using the same luting material, while different lowercase letters in the same row indicate significant differences between luting materials for the same CAD/CAM material. (*p* < 0.05).

**Table 3:** The mean and standard deviations of microhardness (HV) values of the resin luting materials polymerized through CAD/CAM materials.

	Linkforce	G-aenial	Universal Flo
**CONTROL**	47.04 ± 2.08 ^A,b^	56.93 ± 3.84 ^A,a^	57.97 ± 3.05 ^A,a^
**CERASMART**	38.01 ± 4.96 ^B,a^	42.14 ± 1.60 ^B,a^	41.56 ± 3.92 ^B,a^
**VITA ENAMIC**	36.96 ± 2.88 ^B,b^	41.90 ± 2.62 ^B,a^	40.17 ± 2.47 ^B,a^
**CEREC**	39.28 ± 6.01 ^B,a^	42.37 ± 2.34 ^B,a^	42.87 ± 2.68 ^B,a^

Different uppercase letters in the same column indicate significant differences between different CAD/CAM materials using the same luting material, while different lowercase letters in the same row indicate significant differences between luting materials for the same CAD/CAM material. (*p* < 0.05).

## Data Availability

The data presented in this study are available upon request from the corresponding author.

## Abbreviations

The following abbreviations are used in this manuscript:

**CAD/CAM**	computer-aided design/computer-aided manufacturing.
**µTBS**	microtensile bond strength.
**HV**	microhardness.
**SiC**	silicon carbide paper.
**Al_2_O_3_**	aluminum oxide.
**GPB**	G-Premio Bond.
**SEM**	scanning electron microscope.
